# Diet, nutrition and osteoarthritis

**DOI:** 10.1186/1471-2474-16-S1-S7

**Published:** 2015-12-01

**Authors:** Margaret P Rayman

**Affiliations:** 1 Department of Nutritional Sciences, Faculty of Health and Medical Sciences, University of Surrey, Guildford, GU2 7XH, UK

## 

Osteoarthritis (OA) is the fastest growing cause of disability worldwide[1]. In the absence of effective therapies, patients may wish to take some control of their own condition by making dietary changes that have the potential to ameliorate symptoms or reduce disease progression.

A number of dietary factors have been associated with OA symptoms or progression. Most notably, in those overweight, weight reduction of ≥10% has the potential to lead to important changes in pain and function[2]. Losing weight also reduces pain-associated inflammation[3]. Weight loss combined with physical activity has an even greater capacity to improve pain and function[4].

It has been suggested that OA is a metabolic disease in which lipids essentially contribute to the pathophysiology of cartilage degradation[5]. Dietary long-chain ω-3 PUFA may affect articular cartilage composition and appear to have beneficial effects in OA[5]. In a US cohort of individuals with, or at high risk of, knee OA, there was a significant inverse relationship between total n-3 PUFAs and patella-femoral cartilage loss[6].

A positive association has been shown between elevated serum cholesterol and OA; hypercholesterolemia (OR 1.61; 95% CI 1.06-2.47) and high serum cholesterol (3rd vs. 1st tertile: OR 1.73; 95% CI 1.02-2.92) were independently associated with generalized OA in the Ulm study[7]. Hence there may be a potential benefit in adopting dietary cholesterol-lowering strategies (such as consumption of sterol/stanol spreads/drinks).

Vitamin D affects the state of multiple articular structures. The evidence for association between the vitamin D biomarker, serum 25(OH)D, and OA was assessed in a systematic review. For knee radiographic OA progression and cartilage loss, there was strong evidence for an association with low 25(OH)D[8].

Vitamin K is important in cartilage metabolism as an inhibitor of extracellular matrix calcification and a promoter of cell survival/proliferation. In the US MOST study, vitamin K deficiency was associated with incident radiographic knee OA and MRI-based cartilage lesions (RR 2.39; 95% CI, 1.05-5.40) compared with no deficiency[9].

Hence, dietary recommendations are the following:

▪ lose weight, if overweight, preferably combined with exercise;

▪ reduce plasma cholesterol by dietary means;

▪ at least for a trial period, increase intake of long-chain n-3 fatty acids preferably by eating oily fish twice a week;

▪ aim for a safe level of sun exposure, eat rich vitamin-D dietary sources or take vitamin D supplements, ≤ 25 µg/d;

▪ increase vitamin K intake by eating green leafy vegetables.
